# A child feeding index is superior to WHO IYCF indicators in explaining length-for-age Z-scores of young children in rural Cambodia

**DOI:** 10.1179/2046905514Y.0000000155

**Published:** 2015-05

**Authors:** Anika Reinbott, Judith Kuchenbecker, Johannes Herrmann, Irmgard Jordan, Ellen Muehlhoff, Ou Kevanna, Michael Krawinkel

**Affiliations:** 1 Justus Liebig University, Giessen, Germany; 2 The Food and Agricultural Organization of the United Nations, Rome, Italy; 3 National Maternal and Child Health Center, Phnom Penh, Cambodia

**Keywords:** Complementary feeding, Child feeding index, Length-for-age Z-score, Cambodia

## Abstract

**Background:**

Adequate young child feeding practices are influenced by a multitude of factors which affect growth and development. A combination of indicators is needed to explain the role of complementary feeding practices in growth retardation.

**Methods:**

A cross-sectional nutrition baseline survey was conducted in rural Cambodia in September 2012. Villages in pre-selected communes were randomly selected using stunting as a primary indicator. Data were collected from 803 randomly selected households with children aged 6–23 months, based on a standardised questionnaire and on length/height and weight measurements of mother and child. WHO Infant and Young Child Feeding (IYCF) indicators [minimum dietary diversity (MDD), minimum meal frequency (MMF), minimum acceptable diet (MAD)] and a child feeding index (CFI) were created. The latter consisted of five components: breastfeeding, use of bottle, dietary diversity, food frequency and meal frequency which were adjusted for three age groups: 6–8, 9–11 and 12–23 months. The highest possible score was 10. Associations between length-for-age Z-scores (LAZ) and WHO indicators or CFI were explored.

**Results:**

Mean (SD) LAZ was −1.25 (1.14) (*n = * 801). Mean (range) CFI was 6.7 (1–10) (*n = * 797). Mean CFI was highest in the 9–11-months age group (7.93) and lowest for those aged 12–23 months (5.96). None of the WHO IYCF indicators was associated with LAZ, whereas CFI showed significant association with LAZ (*P* < 0.01). The association between higher CFI scores and LAZ became weaker as age increased.

**Conclusion:**

The results highlight the need to include a wide range of information in the analysis in order to understand the association between appropriate infant feeding practices and child growth.

## Abbreviations

CDHS: Cambodian Demographic and Health Survey
CFI: child feeding index
DDS: dietary diversity score
DHS: demographic and health survey
FFQ: food frequency
IYCF: infant and young child feeding
LAZ: length-for-age Z-score
MDD: minimum dietary diversity
MMF: minimum meal frequency
MAD: minimum acceptable diet
WAZ: weight-for-age Z-score;
WLZ: weight-for-length Z-score

## 

## Introduction

Improving the nutritional status of infants and young children by age-appropriate complementary feeding and caring practices by caregivers remains a challenge, especially in low-income countries.^1^ Bhutta *et al.* estimated that scaling-up community-based nutrition approaches aimed at improving infant and young child feeding (IYCF) practices would reduce the overall burden of childhood mortality and also substantially reduce existing disparities.^2^ Various interventions have aimed specifically to improve the diet of children less than 2 years of age as this period is characterised by a rate of high growth and increased vulnerability.^2^,^3^ Hence, inappropriate feeding practices during this critical period can lead to chronic undernutrition and result in stunting.^4^^–^6 Young child feeding practices are influenced by many factors such as maternal health and education, household wealth and food security status. These factors are also known to possibly affect children’s nutritional status.^6^,^7^ Over the years, research has measured and assessed feeding practices in different ways.^8^^–^10 A set of indicators is associated with growth, and a combination of feeding indicators seems to be crucial to provide sufficient information on IYCF practices.^8^,^11^ The IYCF indicators for children aged 6–23 months suggested by the World Health Organization (WHO) include minimum dietary diversity (MDD), minimum meal frequency (MMF) and minimum adequate diet (MAD).^12^ Because of the need for simple, valid and reliable indicators to assess IYCF practices, these three indicators, among others, were created in 2007. They proved to be useful in demographic and health surveys (DHS) in past years. A pooled DHS analysis from 14 countries showed that non-achieved MAD was found to be significantly associated with stunting in children aged 6–23 months whereas there was no relationship between subnormal MMF and stunting.^10^ Using DHS data from five Latin American countries, Ruel & Menon^8^ applied a child feeding index (CFI) that combines single IYCF indicators such as breastfeeding, use of a bottle, dietary diversity (24-hour recall), food frequency (past 7 days) and meal frequency (24-hour recall) to look, amongst others, at associations between child feeding practices and nutritional status. Their results showed significant positive associations between CFI and length/height-for-age Z-scores (LAZ/HAZ) for children aged 6–36 months in four of the five countries. Another study from Senegal showed similar results: LAZ was strongly and positively associated with CFI among infants younger than 1 year of age, though less strongly for 1- and 2-year-old children.^11^

Stunting rates in Cambodia are the second highest of the South-east Asian countries. Data from the 2010 Cambodian DHS (CDHS) showed a 8% decrease in the prevalence of stunting in children aged 0–23 months compared with 2000 (36% in 2000 *vs* 28% in 2010).^14^^–^16 However, stunting remains a major public health issue in the country and is one of the leading causes of morbidity and mortality among children.^17^ It was found to be less likely in infants under 6 months but then increased rapidly up to 12–23 months of age.^18^ According to the 2010 CDHS, 33.5% of Cambodian children aged 6–23 months achieved the MDD, 78.6% MMF and 28.2% MAD.^13^,^15^ In an analysis of DHS data from 14 low-income countries including Cambodia, higher dietary diversity was strongly associated with higher LAZ scores.^10^

The objective of this study was to explore the relationship between feeding practices and LAZ scores in a population of two provinces in north-west Cambodia. The ability of MDD, MMF and MAD to explain length-for-age in this sample was compared with a CFI. The latter was created following Ruel & Menon.^8^ Assuming an interaction of various indicators which reflect usual behaviour of the caregiver, the hypothesis was that the more child feeding recommendations are met, the higher the child’s CFI score and LAZ. However, other factors confounding CFI and LAZ had to be taken into account, such as maternal height, household wealth and the child’s age and gender.^9^

## Methods

A cross-sectional nutrition baseline survey was conducted in mid-September/early October 2012 in collaboration with a food security project of the Food and Agriculture Organization (FAO) of the United Nations. The FAO project includes a component of nutrition education on infant and young child feeding practices linked with components on improving farming systems and building up market links to increase and diversify production, and improve food security amongst smallholding farmers.

### Study sites and study population

The survey was carried out in the 2012 target area of the FAO project in Preah Vihear and Oddar Meanchey provinces. In total, 16 communities from six districts were selected by the project at that time and consequently included in the baseline survey. About 17,650 possible beneficiaries of the FAO project consisting of rural farming households lived in the area. Only households with children aged 0–23 months were eligible to participate. Other inclusion criteria were being resident in the sampled area, being randomly selected, and willingness to participate. After the purpose and following procedure had been explained, informed consent was obtained from each caregiver prior to data collection.

### Design

Using Emergency Nutrition Assessment (ENA) for Smart sample-size calculator and considering a population size of 15,000 children under 2 years of age in the surveyed area, 50% of stunting (primary indicator), a desired precision of ±5% and a design effect of 3, the sample size calculated was 1124 children.^19^ The sampling was conducted using a two-stage probability sampling strategy. Initially, three villages per community were sampled, proportional to population size. At the second sampling stage, 23 households with children aged 0–23 months were randomly selected in each village if more than 23 children in the respective age-range lived in the selected village. If there were exactly 23 children of the required age range, all caregiver–child pairs were asked to participate. If the village was very small and had fewer than 23 suitable children, eligible households were selected at random in the nearest adjacent village to complete the required sample.

Overall, 1028 households with a child between 0 and 731 days^i^ (under 2 years of age) participated in the survey. For this analysis, only children between 6 and 23 months and thus at the complementary feeding age were considered, resulting in a final sample size of 803 households.

### Data collection procedure in the field

In each village, the selected primary caregivers with their children were invited to a central meeting point to participate in the survey. The children’s ages were verified at this point by cross-checking the birth dates indicated on village lists with the vaccination cards or birth certificates. In seven cases, where there was no information on the child’s age, the age was estimated using a local events calendar and later dated to the 15th of the named month.

Semi-structured questionnaires included a household, child and caregiver section and were administered in face-to-face interviews with the primary caregiver of the under 2-year-old child in the selected household. Data collected included socio-economic and demographic information on the household, household and child dietary diversity based on 24-hour recall, child’s 7-day food frequency, feeding and caring practices and hygiene practices. In addition, episodes of fever, diarrhoea and cough were assessed for the 2 weeks before the survey, as perceived by the caregiver. Anthropometric measurements were taken from the mother and child with standardised equipment from Seca (Seca GmbH & Co. KG, Hamburg, Germany): digital flat weighing scales with mother/child function (Seca 874, capacity 200 kg, SECA, Germany; kg to two decimal points), lengthboards (Seca 417, measurement range 10–100 cm, SECA, Germany), and stadiometers (Seca 213, measuring range 20–205 cm, SECA, Germany). Mothers’ heights and weights were collected as well as the childrens’ lengths and weights, following the FANTA protocol. Height/length and weight were assessed to the nearest 0.1 cm and 0.1 kg, respectively.^20^ All measurements were taken twice. The maximum tolerated difference between the two measurements was 1.0 cm for height/length and 0.5 kg for weight.^20^ The mean of both measurements was used for the final analysis.

All data collection tools were pre-tested in the field. Quality control of the data collection was conducted regularly.

### Wealth index

Socio-economic data were used to develop a wealth index based on the results of a principal component analysis. Variables included in the wealth index were number of persons per rooms used for sleeping, floor composition, type of sanitation facility, drinking water source and ownership of land and certain assets (e.g. radio, television, mobile and non-mobile phone, wardrobe, sewing machine or loom, CD/DVD player, generator/battery/solar panel, watch, bicycle, motorcycle, motorcycle-cart, car/truck/van, boat, ox-/horse-cart and hand-tractor).^21^,^22^

### Indicators for infant and young child feeding and child’s nutritional status

Feeding practices were assessed using the following WHO IYCF indicators for children aged 6–23 months: continued breastfeeding, introduction of solid, semi-solid and soft foods, MDD, MMF and MAD.^12^,^13^ These indicators look at the percentage of children meeting the recommended criteria.

The CFI was created for children aged 6–23 months following Ruel & Menon and Arimond & Ruel.^8^,^9^ It consists of five different components based on current national and international young child feeding recommendations^23^ (Table 1). Scoring points were given when the child was still breastfed and not bottle-fed.^ii^ Dietary diversity score (DDS) based on 24-hour recall emphasised six different food groups which resulted in a maximum of six scoring points. Food frequency (FFQ) reflected the consumption of certain foods in the past 7 days, which were then totalled in a combined score. Meal frequency assessed the intake of solid, semi-solid and soft foods in the past 24 hours and resulted in scoring points for a certain number of meals given. DDS, FFQ and meal frequency were matched to the different requirements for each age group (6–8.9, 9–11.9 and 12–23.9 months), as indicated in Table 2. The CFI could reach a maximum of 10 scoring points. Following Ruel & Menon and others, the index was treated as a continuous variable.^8^,^24^,^25^ However, most other publications mainly use and present results from models with the CFI included as a dichotomous variable or as terciles rather than presenting means or regression models, emphasising that the CFI used as a categorical variable is useful for graphic models and a strong advocacy tool.^9^,^11^,^25^ In this study, creating terciles would have led to a loss of information.

**Table 1:** Current infant and young child feeding recommendations in Cambodia^23^

6–8 months	9–11 months	12–23 months
Continue breastfeeding (8 times/day)	Continue breastfeeding (6 times/day)	Continue breastfeeding (on demand)
Cup feeding (no bottle)		
2–3 meals per day	3 meals/day	3 meals/day
Gradually increase	½–1 bowl/meal	1 bowl/meal
amount per meal		
from 2 tablespoons		
to ½ bowl		
	1 snack/day	2 snacks/day

**Table 2:** Scoring system for child feeding index* by age group

	6–8 months		9–11 months		12–23 months	
Breastfeeding	NoYes	= 0 = +2	NoYes	= 0 = +2	NoYes	= 0 = +1
Use of bottle	NoYes	= +1 = 0	NoYes	= +1 = 0	NoYes	= +1 = 0
Dietary diversity (past 24 hrs)	*Sum of:* (grains/tubers + meat/fish + eggs + legumes + vitamin A-rich fruits and vegetables + other fruits/veg.)		*Sum of:* (grains/tubers + meat/fish + eggs + legumes + vitamin A-rich fruits and vegetables + other fruits/veg.)		*Sum of:* (grains/tubers + meat/fish + eggs + legumes + vitamin A-rich fruits and vegetables + other fruits/veg.)	
	01–34+	= 0 = +1 = +2	01–34+	= 0 = +1 = +2	01–34+	= 0 = +1 = +2
Food frequency (past 7 d)	*For egg/fish/meat*0 times in past 7 d = 01–3 times in past 7 d = 14 times in past 7 d = 2*For staples (grains or tubers)*0–2 times = 0; 3+ times = 1		*For egg/fish/meat*0 times in past 7 d = 01–3 times in past 7 d = 14 times in past 7 d = 2For staples (grains or tubers)0–3 times = 0; 4+ times = 1		*For each of milk and egg/fish/meat*0 times in past 7 d = 01–3 times in past 7 d = 14 times in past 7 d = 2	
	*Food frequency* = sum of scores (egg/fish/meat + staples)		*Food frequency* = sum of scores (egg/fish/meat +)		*Food frequency* = sum of scores (egg/fish/meat + milk)	
Meal frequency† (past 24 hrs)	0 meals/d1 meal/d2 meals/d	= 0 = 1 = 2	0 meal/d1–2 meals/d3+meals/d	= 0 = 1 = 2	0–1 meal/d2–3 meals/d4+meals/d	= 0 = 1 = 2
Total score	10 points		10 points		10 points	

*Adjusted by the authors based on Ruel & Menon;^8^

^†^ meal frequency does not include breast-milk or any other liquids and only refers to solid, semi-solid or soft foods received in the past 24 hours

### Statistical analysis

All data were entered into EpiData (version 3.1) twice and analysed by SPSS (IBM, SPSS Statistics version 20.0.0.2).^26^,^27^ Before testing for associations between different indicators and LAZ, the data were tested for dependencies, intra-class correlations and clustering effects between the different regions (e.g. provinces, districts and communities).^28^ No considerable clustering effects were seen in the sample. Heteroscedasticity was precluded as both the Koenker test and the Breusch-Pagan test were not significant.^29^

To compare mean LAZ values between different groups (e.g. gender, IYCF indicators achieved *vs* not achieved), independent *t*-tests as well as one-way ANOVAs were performed. Linear regression models were used for several continuous variables such as age, height/length, education in years and wealth index, to act as covariates to test their association with LAZ scores. Bivariate correlations using Pearson’s r between CFI components and LAZ by age group were performed.

Correlations between the child’s age and the different CFI components for each different CFI age group were explored. To determine the degree of association between each of the CFI components and LAZ scores, bivariate correlations were undertaken, stratified by CFI age group. Regression analysis was performed with LAZ as the dependent variable and CFI as the independent variable. In accordance with Ruel & Menon, the following covariates were included in the model: age and gender of the child, age, height, BMI and education in years of the mother, wealth index and the number of children below 2 years of age in a household.^8^ Besides linear regression, an additional non-linear regression analysis (quadratic model) was conducted. Univariate analysis was performed with and without covariates. Estimates of marginal means were calculated for each CFI scoring point. Moderator and mediator models were applied to assess the role of different moderators and mediators on the relationship between CFI and LAZ.

## Results

### Background characteristics of the households

The main household and child characteristics are presented in Table 3. On average, the 803 households each had five members. Literacy was poor amongst caregivers (53%). Unimproved sanitation facilitiesiii were common in 82% of the households whereas only 13% had an unprotected source of drinking water^iv^. The nearest health facility was within 1 hour’s reach of 87% of the households. The diet of a household mainly consisted of a variety of between five to eight food groups (75%, maximum 12 food groups). There were no significant correlations between homegarden ownership, wealth and household dietary diversity in the sample.

**Table 3:** Selected household characteristics, WHO IYCF indicators and child nutritional status

	%	Mean	SD	Range
No. per household (*n* = 803)		5.09	1.86	2–13
No. of children <2 yrs (*n* = 803)		1.03	0.17	1–3
Literacy rate respondents (*n* = 803)	52.8			
Main income sources (*n* = 803)				
Agriculture	70.7			
Employment/salary	14.4			
Homegarden ownership (*n* = 803)	70.4			
Household grows vegetables (*n* = 803)	63.5			
Sanitation facilities (*n* = 803)				
Improved	18.3			
Unimproved	81.7			
Drinking water source (*n* = 803)				
Protected	86.6			
Unprotected	13.4			
Household dietary diversity score (*n* = 803)		6.78	1.72	2–12
WHO indicators achieved				
Minimum dietary diversity (*n* = 801)	43.9			
Minimum meal frequency (*n* = 773)	69.9			
Minimum acceptable diet (*n* = 798)	28.3			
Mean child dietary diversity score (*n* = 798)		3.24	1.50	0–7
Child’s nutritional status				
Mean length-for-age Z-score (*n* = 801)		−1.25	1.14	−5.82–4.15
Prevalence of stunting (<−2 SD LAZ)	25.1			
Mean weight-for-length Z-score (*n* = 802)		−0.77	1.03	−4.04–4.54
Prevalence of wasting (<−2 SD WLZ)	9.7			
Mean weight-for-age Z-score (*n* = 803)		−1.22	1.04	
Prevalence of underweight (<−2 SD WAZ)	22.8			−5.44–2.88

### Main characteristics of the children

Of the 803 children, 447 were male (56%). The mean age (range) was 14 months (6-24). The majority were born in a health facility and/or with the attendance of trained health staff (76%) and were fully vaccinated according to the WHO definition (88%). During the 2 weeks before the survey, 69% suffered from fever, 39% from diarrhoea and 5% from acute respiratory infections (ARI), as expected during the wet season.

### Infant and young child feeding practices

Almost all the 803 children had been breastfed at some time (99.8%) and were still breastfed at the time of the survey (82%). Breastfeeding was continued for 93% of 186 children aged 12–15 months. Solid, semi-solid or soft foods were introduced to 94% of the 163 children between 6 and 8 months. The diet of all the children consisted on average of 3.2 food groups (maximum seven food groups). Overall, MDD was achieved by 44%, MMF by 70% and MAD by 28%.^13^

The CFI showed a mean (range) score of 6.7 (1–10) (*n = * 797). Mean CFI was highest in those aged 9–11 months (7.9, *n*  =  169), followed by those aged 6–8 months (7.5, *n* =  158). Children between 12 and 23 months of age achieved a mean score of 6.0 (*n*  =  417) (Table 4). Bottle feeding was most prevalent among the 6–8-month-olds. Liquids fed in a bottle were mainly water (74%) and infant formulae (17%). Breastfeeding showed a decline after 12 months of age. Diet in the two younger age groups consisted mainly of three food groups and changed to three or four food groups in the 12–23-month-olds. An analysis of the correlation between the components of the CFI and age showed that, for children aged 6–8 months, DDS, FFQ and meal frequency increased significantly with age: *r* =  0.256, *r* =  0.284 and *r* =  0.325, respectively (all *P* < 0.001). Among the 12–23-month-olds, age and DDS, FFQ and meal frequency correlated significantly but less strongly so. There were no significant correlations between age and one of the CFI components among the children aged 9–12 months. The number of breastfed children decreased with age among children over 12 months (*r* −0.463, *P* < 0.001).

**Table 4:** Child feeding index (CFI) components by CFI age-group

Component	6–8 months *n* = 158		9–11 months *n* = 169		12–23 months *n* = 417	
Breastfeeding, %	98.8		95.2		71.3	
No bottle used, %	43.6		39.5		27.3	
Dietary diversity score, %						
Low	0–2 groups:	6.1	0–2 groups:	4.7	0–2 groups:	1.5
Medium	3 groups:	73.6	3 groups:	59.2	3 groups:	48.2
High	≧4 groups:	20.2	≧4 groups:	36.1	≧4 groups:	50.3
Food Frequency						
Range	0–3		0–3		0–4	
Median	2		3		2	
Mean (SD)	2.08 (1.1)		2.44 (0.9)		1.96 (0.8)	
Meal frequency, %						
Low	0 meals/d:	6.2	0 meals/d:	3.0	0–1 meal/d:	6.4
Medium	1 meal/d:	11.7	1–2 meals/d:	25.4	2–3 meals/d:	79.8
High	2 meals/d:	82.1	≧3 meals/d:	71.6	≧4 meals/d:	13.8
CFI						
Range	2–10		2–10		1–10	
Median	8		8		6	
Mean (SD)	7.52 (1.8)		7.93 (1.6)		5.96 (1.2)	

### Nutritional status

**Figure 1:**
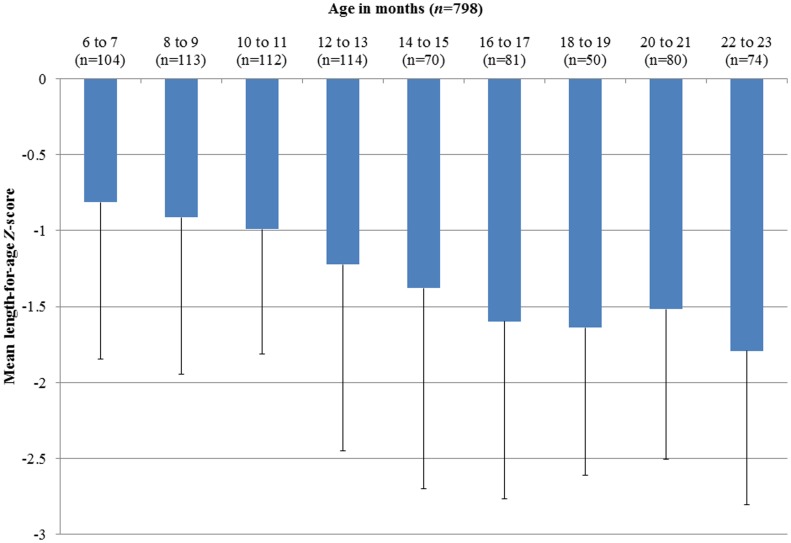
Mean (SD) LAZ per 2-months age-group (*n* =  798). (The error bars show −1 SD of the mean).

Mean (SD) LAZ score was −1.25 (1.14) for all children aged 6–23 months (*n*  =  801), and did not differ significantly between genders. Overall, 25% of the children were stunted with an LAZ score below −2 SD, and 4% were severely stunted with a LAZ score below −3 SD. Weight-for-age Z-scores (WAZ) below −2 SD were recorded for 23% (*n*  =  803). Prevalence of wasting was 10%, as shown by weight-for-length Z-scores (WLZ) below −2 SD, and 1.2% of them were severely wasted (*n*  =  802). Five per cent of the children were stunted and wasted. Mean LAZ scores decreased by age as shown in Figure 1.

### Association between LAZ and different indicators

Non-nutritional parameters were associated with LAZ such as the assistance of a health professional at birth or whether the caregiver had ever attended school; both showed a weak correlation (*r * = 0.07, *P*  =  0.04; *r* =  0.07, *P*  = 0.05). Also the correlation with household’s wealth index was weak (*r * =  0.06, *P* =  0.088). Stronger correlations were found between LAZ and the child’s age (*r * =  0.28, *P* < 0.001) and maternal height (*r* =  0.27, *P* < 0.001). Nutritional indicators such as breastfeeding, MDD, MMF and MAD were either not or only weakly correlated with LAZ scores for all children (Table 5) and the correlation remained weak and non-significant for different age groups (6–8, 9–11 and 12–23 months).

**Table 5:** Associations of WHO IYFC indicators and LAZ - results from independent sample t-test

		*n*	Mean LAZ	SD	SE (mean)	t (dF)	*P*	95% CI LAZ difference
Minimum dietary diversity (6–23 mths)	Yes	351	−1.28	1.05	0.06	0.68 (788)	0.49	−0.10, 0.21
	No	448	−1.22	1.20	0.06			
Minimum meal frequency (6–23 mths)	Yes	538	−1.21	1.16	0.05	−1.42 (769)	0.16	−0.30, 0.49
	No	233	−1.34	1.09	0.07			
Minimum acceptable diet (6–23 mths)	Yes	225	−1.21	1.03	0.07	−0.50 (794)	0.62	−0.22, 0.13
	No	571	−1.26	1.18	0.05			

Children with an LAZ score below −2 SD had a lower CFI score [mean (SD) 6.4 (1.5), *n*  =  198] than those with LAZ scores above −2 SD [mean (SD) 6.8 (1.8), *n* =  597]. This difference was significant: *t* (386.6) 2.95, *P*  = 0.003 with an effect size of *r* = 0.181.

Table 6 shows the correlation between the different CFI components and LAZ and not only between FFQ and LAZ, stratified by age group. FFQ was significant for the two age groups below 1 year only. LAZ of the children aged 12–23 months was not associated with any particular CFI indicators.

**Table 6:** Bivariate correlations between CFI components and LAZ by age group

Age group, mths	Breastfeeding		No bottle used		DDS		FFQ		Meal frequency	
	r*	*n*	r*	*n*	r*	*n*	r*	*n*	r*	*n*
6–23	0.189†	799	−0.035	797	−0.038	801	0.136†	801	0.140†	800
6–8	−0.019	163	0.048	163	0.097	163	0.189‡	163	0.046	162
9–11	−0.126	167	0.093	167	0.030	169	0.202†	169	0.032	169
12–23	0.003	469	−0.061	467	0.003	469	0.005	469	−0.050	469

*Pearson’s *r*;

^†^correlation is significant at the 0.01 level (2-tailed);

^‡^correlation is significant at the 0.05 level (2-tailed).

**Figure 2:**
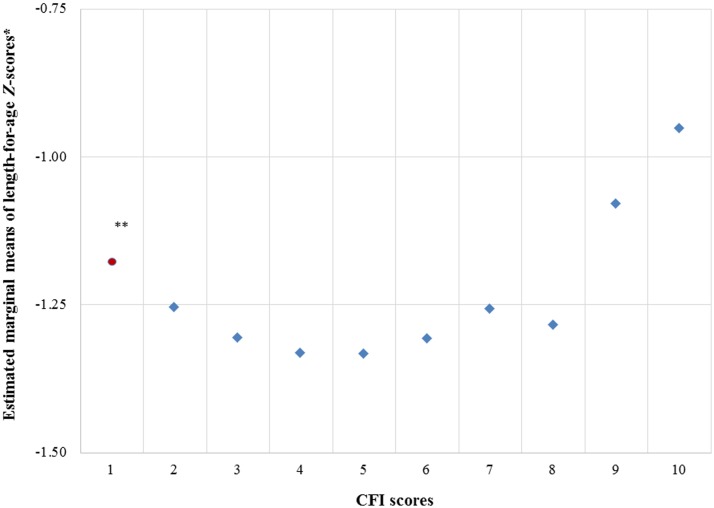
Estimated marginal means of LAZ scores by CFI scores. No. of cases: CFI 1 =  1, CFI 2  =  7, CFI 3 =  25, CFI 4  =  35, CFI 5 =  105, CFI 6  =  201, CFI 7 =  189, CFI 8  =  106, CFI 9 =  83, CFI 10  =  45. * Covariates appearing in the model are evaluated at the following values: CFI, CFIsq, age (days), household members aged <2 years, education respondent (years of schooling), household’s wealth index, age of mother, height (cm) of mother, BMI of mother, sex of child; ** CFI score 1 represents one case only, thus the estimation might be biased. (See Table 1 in supplementary material for the exact value of the estimated marginal means, standard error and 95% confidence intervals, www.maneyonline.com/doi/suppl/10.1179/2046905514Y.0000000155.)

A linear regression model including specific child, maternal and household characteristics in the model showed significant positive correlation between CFI and LAZ (R^2^  =  0.156, B =  0.051, b  =  0.077, *P*  =  0.04). Maternal height and child’s age were shown to be significant in influencing the association between CFI and LAZ, whereas the child’s gender, maternal age, maternal BMI, maternal education, household wealth and number of children aged 0–23 months in the household were not significantly associated. A regression model with only the two covariates which reached significance, age of the child and maternal height, did not show a stronger association between LAZ and CFI than the previous model with more than these two covariates. Since the assumption of linearity was not met, a quadratic model (CFI-sq) was considered more suitable for explaining the data (excluding the one child with CFI 1): from CFI 2 to CFI 4, the mean LAZ levels decreased and remained at a similar level until CFI 6. Above CFI 6, mean LAZ levels increased, apart from a drop at CFI 8 (Fig. 2). Although the number of children with CFI scores 1–4 was small, two quadratic models with hierarchic regression were applied, one without and one without covariates. Both CFI and CFI-sq were significantly associated with LAZ, without including covariates in the model (b_CFI_ -0.3, b_CFI-sq_ 0.03). After including covariates in the model, CFI and CFI-sq were no longer significantly associated with LAZ, but the model showed a more linear and weaker association (b_CFI_ −0.1, b_CFI-sq_ 0.01). Thus, for further analyses, the CFI was disaggregated into two groups: CFI_1–4_ and CFI_5–10_.

The relationship between LAZ and CFI was affected by the child’ age. Results from a moderator analysis showed significant interaction between CFI_5–10_ and LAZ with age as a moderator (Table 7). The conditional effect of CFIs of 5–10 on LAZ at different values of the moderator *age* shows that CFI_5–10_ has a significant positive effect (effect 0.09, *P*  =  0.02) on LAZ under the condition that children’s age is the mean of age −1 SD (1 SD 157 days). At mean age and older (mean age +1 SD), the effect becomes non-significant and even negative for the oldest age group (effect − 0.04).

**Table 7:** Linear model of predictors of LAZ disaggregated into CFI_1–4_ and CFI_5–10_

CFI		b	SE B	*t*	*P*
1–4 (*n* = 67)	Constant	−4.64	3.98	−1.17	0.25
	Age in days	−0.0001	0.0008	−0.16	0.87
	CFI	−0.45	0.18	−2.49	0.02
	Age in days × CFI	0.0004	0.0013	0.33	0.75
5–10 (*n* = 696)	Constant	−10.05	1.13	−8.89	0
	Age in days	−0.002	0.0003	−6.52	0
	CFI	0.03	0.03	0.76	0.45
	Age in days × CFI	−0.0004	0.0002	−1.99	0.05

Wealth had a slight effect on LAZ through the CFI_1–4_ as mediator (b −0.016, SE (b) 0.013, 95% CI (b) −0.052–0.002, κ^2^  =  0.048). The effect decreased for CFI_5–10_: b 0.007, SE (b) 0.0031, 95% CI (b) 0.002–0.014, κ^2^  =  0.020.^31^

## Discussion

A combination of indicators for young child feeding reflected in the CFI, as suggested by Ruel & Menon,^8^ was found to be significantly associated with LAZ. The more that recommended criteria were met by caregivers, the more likely it was that the 6–23-month-olds achieved age-appropriate length. In contrast, the caregivers’ practices assessed by the WHO IYCF indicators did not explain the observed decrease of LAZ with age. Other variables known to possibly affect LAZ, such as household’s socio-economic status, access to improved sanitation facilities and maternal education, were not associated with LAZ.^5^,^32^,^33^

However, if stratified by age group, the association between CFI and LAZ was significant only for children under 1 year. The older the child, the less a CFI score above 5 was associated with LAZ. This supports findings from a prospective open-cohort study by Bork *et al.* who also described a decreasing association of CFI with LAZ with age.^11^ Their sample (*n*  =  500) from Senegal showed strong positive associations among children aged 6–12 months only (*P* < 0.001). Adjustments for wealth, maternal height, education and occupation did not change their results which conform partly to our findings in which wealth, maternal education and occupation also did not significantly influence the relationship between CFI and LAZ.^11^ Another study from Shanghai used a slightly different CFI which showed no significant association with LAZ for 12-month-old children, but an association for 6- and 18-month-old children.^24^ Their findings are based on a longitudinal study design following 180 children for 12 months, starting with 6-month-old children. They finally concluded that CFI might be used to evaluate the effects of child feeding on growth for a longer time.^24^

Associations between LAZ and CFI were also investigated by Ruel & Menon in Latin America (2002, DHS data) and by Sawadogo *et al.* in Burkina Faso (2006, *n*  =  2466 children and 2411 mothers).^8^,^25^ Both described strong correlations between LAZ and CFI; Bork *et al.* in Senegal (2012, *n* =  500) and this study (*n* =  803) prove the association only in younger children.^11^

Causes of stunting other than those reflected by the CFI need to be considered. Whether the negative associations between CFIs and LAZ in any age-sub groups are related to neglected components of infant and young child feeding practices in the different CFIs used or are explained by poor statistical power needs further investigations.

The highest breastfeeding rates in this Cambodian study population were in the 6–8-month-olds, a subgroup in which the use of bottles is highly prevalent too. Those figures accord with the latest CDHS which reported that 82% of 6–8-month-olds are still being breastfed and that 27% of the same age group are bottle-fed.^15^ In this study, the children of 12–23 months had lower CFI scores than infants, mainly because of low food frequency and low breastfeeding rates. This age group also showed the lowest mean LAZ score (−1.50). This contradicts Arimond’s & Ruel’s general conclusion of a strong positive association between LAZ and dietary diversity.^34^ In this Cambodian sample, LAZ scores were not associated with dietary diversity in all age groups, whereas LAZ scores were positively correlated with higher food frequency, particularly in children aged 6–11 months. However, Arimond & Ruel analysed nationally representative samples taken from DHS surveys which presumably showed a greater diversity of dietary practices.

In rural Cambodia, where knowledge of adequate complementary feeding practices is poor, children do not receive an age-appropriate diet, aside from the practice of exclusive breastfeeding of children under six months of age. The main reasons for poor complementary feeding practices for children from 6 months of age onwards are poor quality of the meal, especially low energy and nutrient density, and inadequate feeding frequency.

Just as complementary feeding practices have a wide range of characteristics and measurement approaches, growth faltering resulting in stunting has various causes which cannot be determined by assessing only complementary feeding practices. Growth faltering in children under 2 years of age is mainly the result of interaction between intrauterine growth retardation, suboptimal breastfeeding, micronutrient deficiencies, reduced energy intake, infection, sanitation facilities and other factors related to poverty.^1^

In this study, the relationship between household wealth and LAZ was less strong than in studies by Hong & Mishra and Marriott *et al.* who both analysed CDHS data.^18^,^35^ Hong & Mishra concluded that children in comparatively poorer households are at much greater risk of suffering chronic undernutrition than children in wealthier households.^18^ Marriott *et al.* concluded that wealth calculated as an asset-based family wealth index was consistently associated with a lower probability of being stunted.^35^

In Cambodia, the diversity of complementary foods for infants is usually poor. The predominant porridge is made from rice and water and is of low nutritional value. Most of the children received fish and vegetables from 12 months of age onwards.^37^ Jones *et al.* proposed including different indicators of food quality, safety and feeding behaviour to analyse child feeding practices.^38^ Another conclusion could be that changes in feeding practices only influence LAZ above a certain minimum level as there was no difference for feeding less than three different food groups.

The history of breastfeeding and its possible impact on LAZ should also be taken into account. Results from a cross-sectional study in Malawi showed a significant association between exclusive breastfeeding and LAZ.^39^ In Cambodia, which has much higher exclusive breastfeeding rates, the initiation of breastfeeding and place of delivery might be relevant to the subsequent child feeding pattern. The latter in particular offers access to information on IYCF practices.

A study in India used a different young child feeding index.^40^ Their index consisted of the age when solid, semi-solid and soft foods were introduced, the minimum amount per meal, and active feeding practices as well as consistency and safety of food in addition to breastfeeding, bottle feeding, meal and food frequency and dietary diversity. This resulted in a score which was significantly higher for well nourished than for undernourished children aged 6–23 months, as found in this study for stunted *vs* not stunted children.

This study has limitations owing to its cross-sectional design and the difficulty of making assumptions regarding long-term impact. Further research is needed to also investigate seasonal differences in child feeding and its impact on the child’s nutritional status. Also, child development such as growth and motor skills might influence mothers’ feeding responses and behaviour. As the effect of breastfeeding on child development has been well demonstrated, whether a child was exclusively breastfed before complementary feeding commenced at 6 months of age also needs to be considered. Thus, the general variable ‘breastfeeding’ should be differentiated by including the time when breastfeeding is initiated and the use of pre-lacteal/early supplementary feeds.

The objective of the study was to analyse the relationship between IYCF practices and LAZ scores in rural Cambodia. Findings demonstrate that the age of the child and maternal height were significantly associated with LAZ scores. The WHO IYCF indicators showed no strong or significant association with LAZ. The composite CFI was weakly associated with LAZ scores of children aged 6–23 months. Statistical significance was demonstrated only in the younger children. The correlation between CFI and LAZ, however, was unexpectedly negative for CFI 1–4 and became positive for CFI 5 and higher, suggesting a lack of relevance of differentiation for CFI less than 5. Overall, the CFI was therefore considered to be more useful in explaining the link between IYCF practices and LAZ in this sample than the more simple WHO IYCF indicators.

Although this study demonstrated associations between a composite CFI and LAZ in infants and young children, more research is required to assess IYCF practices with regard to nutrition education. Even where local resources allow for adequate infant and young child feeding, nutritional knowledge and awareness, and factors such as mothers’ available time are crucial to improving the nutritional status of the children.

## Disclaimer Statements

**Contributors** AR assessed, analysed and interpreted the data. She drafted the manuscript with the contribution of MK and I J who designed the overall study. JH proofed the data analysis and contributed to the manuscript. JK assisted during data collection and contributed to the manuscript. EM advised the research team on the study design and contributed to the development of the questionnaire as well as the manuscript. OK was the national principal investigator in Cambodia; MK was the principal investigator of the overall IMCF study.

**Funding** The study was funded by the Food and Agricultural Organization of the United Nations with support of the German Federal Ministry of Food and Agriculture.

**Conflicts of interest** None.

**Ethics approval** The study was approved by the Institutional Review Board of Justus Liebig University and the National Ethics Committee for Health Research in Cambodia, and registered at the German Clinical Trials Register (no. DRKS00004379).

## Footnotes

^i^As 2012 was a leap year with 366 days, children aged 731 days were counted as children aged 0–23 months.

^ii^No bottle feeding does not mean no liquids were given; liquids besides breast-milk are not considered in this index. Bottle feeding is regarded as harmful mainly for reasons of hygiene.

^iii^Sanitation facilities as classified by UNICEF/WHO: improved =  facilities that ensure hygienic separation of human excreta from human contact; unimproved  =  facilities that do not ensure hygienic separation of human excreta from human contact^30^

^iv^Source of drinking water as classified by UNICEF/WHO: protected/improved  =  sources that, by nature of their construction or through active intervention, are protected from outside contamination, particularly faecal matter; unprotected/unimproved =  unprotected dug well, unprotected spring, cart with small tank/drum, tanker truck, and surface water, bottled water.^30^
